# Prospective Randomized Study on the Influence of Myoinositol in PCOS Women Undergoing IVF in the Improvement of Oocyte Quality, Fertilization Rate, and Embryo Quality

**DOI:** 10.1155/2016/4378507

**Published:** 2016-08-22

**Authors:** Bernd Lesoine, Pedro-Antonio Regidor

**Affiliations:** ^1^Centre for Reproductive Medicine, Prinzregentenstraße 69, Bogenhausen, 81675 Munich, Germany; ^2^Frauenklinik München West, Schmiedwegerl 2-6, 81241 Munich, Germany

## Abstract

Polycystic ovarian syndrome (PCOS) is one of the pathological factors involved in the failure of in vitro fertilization (IvF). The aim of the present study was to investigate if the combination of myoinositol + folic acid was able to improve the oocyte quality, the ratio between follicles and retrieved oocytes, the fertilization rate, and the embryo quality in PCOS patients undergoing IvF treatments. 29 patients with PCOS underwent IvF protocols for infertility treatment and were randomized prospectively into two groups. Group A (placebo) with 15 patients and group B (4000 mg myoinositol + 400 *μ*g folic acid per day) with 14 patients. The patients of group B used for two months myoinositol + folic acid before starting the IvF protocol and data were obtained concerning number of follicles, number of oocytes, quality of oocytes, fertilization rates, and embryo quality in both groups. The ratio follicle/retrieved oocyte was better in the myoinositol group (= group B). Out of the 233 oocytes collected in the myoinositol group 136 were fertilized, whereas only 128 out of 300 oocytes in the placebo group were fertilized. More metaphase II and I oocytes were retrieved in relation to the total amount of oocytes in the myoinositol. More embryos of grade I quality were obtained in the myoinositol. The duration of stimulation was 9,7 days (±3,3) in the myoinositol group and 11,2 (±1,8) days in the placebo group and the number of used FSH units was lower in the myoinositol group: 1750 FSH units (mean) versus 1850 units (mean). Our evidence suggests that myoinositol therapy in women with PCOS results in better fertilization rates and a clear trend to a better embryo quality. As the number of retrieved oocytes was smaller in the myoinositol group, the risk of hyper stimulation syndrome can be reduced in these patients.

## 1. Introduction

The Polycystic Ovary Syndrome (PCOS) is a disease that causes irregular bleeding, chronic anovulation, androgen excess, and a typical ovarian ultrasound feature [1]. It is the most common cause of infertility affecting between 5% and 10% of women during their reproductive age [2]. The etiology of the syndrome is still unknown. The European Society of Human Reproduction and Embryology and the American Society for reproductive Medicine defined at a meeting in Rotterdam 2003 the criteria for the definition of this disease [3, 4]. In order to reach a general agreement on diagnostic criteria many investigations have focused, independently of the above-mentioned disorders, on the impaired glucose tolerance, which affects 30–40% of women with PCOS [5], and on insulin resistance. Insulin resistance is common in PCOS women, regardless of the body mass index (BMI). Hyperinsulinemia due to insulin resistance occurs in up to 80% of women with PCOS and central obesity as well as in 30–40% of lean women diagnosed with this syndrome [6, 7].

Studies have suggested that an impairment of the insulin pathway could be due to a defect in the inositol phosphoglycans (IPGs) second messenger [8, 9]. IPGs play a role in activating enzymes that control glucose metabolism [10, 11]. In PCOS women, a defect in tissue availability or altered metabolism of inositol or IPGs mediators may contribute to insulin resistance [12]. Previous studies have demonstrated that myoinositol is able to restore spontaneous ovarian activity in PCOS women and consequently fertility in many of these cases [13]. Many of these PCOS women require techniques of assisted reproduction to achieve a pregnancy. However, more than 60% of in vitro fertilization (IVF) cycles do not result in a pregnancy and poor oocyte quality is the main cause of fertilization failure in assisted reproductive techniques [14]. Therefore assisted reproductive techniques nowadays focus on obtaining high quality oocytes rather than high numbers of oocytes and embryos [15]. In IVF techniques, the supplementation with myoinositol is positively correlated to a meiotic progression of mouse germinal vesicle oocytes, enhancing intracellular Ca2^+^ oscillations [16]. In follicular human fluids, higher concentrations of myoinositol represent a marker of good-quality oocytes [17].

Whether at the end myoinositol or D-Chiro-Inositol has a better effect on the quality of the oocytes in IVF cycles has to be elucidated. The paradox theory published and discussed by Nestler and Carlomagno [18, 19] suggests that D-Chiro Inositol in not physiological high dosages has a negative impact on the quality of oocytes of PCOS women, as the ovarian epimerase function is not altered in PCOS patients so that the myoinositol levels in the follicular fluids remain low if only D-Chiro-Inositol is supplemented. The Consensus Conference on myoinositol and D-Chiro-Inositol postulated that the future ideal inositol supplementation should therefore contain a ratio of 40 : 1 between myoinositol and D-Chiro-Inositol [20].

The approach of myoinositol with 3 mg melatonin is also a way in the possible improvement of oocyte quality. Rizzo et al. [21] and Unfer et al. [22] could obtain oocytes with a better quality when the PCOS women were treated in combination with myoinositol 4 grams + 400 *μ*g folic acid + 3 mg melatonin per day in comparison to formulations without melatonin.

The aim of the present study was to investigate if pretreatment with only myoinositol and folic acid as a food supplement was able to improve the oocyte quality, the ratio between follicles and retrieved oocytes, the fertilization rate, and the embryo quality in PCOS patients undergoing IVF treatments. Due to the paradox theory, D-Chiro-Inositol was not used and melatonin 3 mg per day was excluded because of its drug status in Germany.

## 2. Materials and Methods

### 2.1. Patients

Twenty-nine patients with PCOS underwent IVF protocols for infertility treatment in the Centre for Reproductive Medicine Bogenhausen in Munich, Germany, and were randomized prospectively into two groups. Group A (placebo) with 15 patients and group B (4000 mg myoinositol + 400 *μ*g folic acid per day) with 14 patients were evaluated. The patients of group B used for two months a combination of myoinositol and folic acid before starting the IVF protocol. The patients aged < 40 with PCOS indicated by oligomenorrhoea and/or hyperandrogenism and/or hyperandrogenemia and/or typical features of ovaries on ultrasound scan were enrolled in this study. At least two of the above-mentioned criteria were present in all the patients. The women had no other medical conditions causing ovulatory disorders such as hyperprolactinemia or thyroidal disorders or Cushing syndrome.

### 2.2. Controlled Ovarian Hyperstimulation

In all patients, the stimulation was started on cycle day 2 or 3 with a single dose of 150 IU gonadotropin ß. All patients received from the 6th stimulation day the antagonist Orgalutran® (Ganirelix) to prevent a premature ovulation.

Ovulation induction was performed on all the patients on day 10 or 11 of the stimulation either with 5000 IU Brevactid® (hCG), or in case of risk of an overhyperstimulation syndrome with 0.2 mg Decapeptyl® (GnRH-agonist).

Follicular puncture was performed on all patients exactly 35 hours after induction of ovulation. Those patients at risk of an overstimulation syndrome did not received an embryo transfer but the fertilized oocytes were cryoconserved at the preembryonic stage. Embryo transfer (maximum 2 embryos) was performed after 2, 3, or 5 days. Pregnancy tests were performed always 14 days after follicular puncture.

### 2.3. Luteal Phase

Vaginal administration of 400 mg micronized progesterone was started on the day of ovum pick-up and treatment was continued until either a serum pregnancy test result was negative or an embryonic heart was sonographically confirmed.

Data were obtained concerning number of follicles, number of retrieved oocytes, ratio follicles/oocytes, quality of oocytes, fertilization rates, and embryo quality.

For statistically analyses a Student's *t*-test was performed to compare the effects between the treatment groups.

## 3. Results

**Figure 1:**
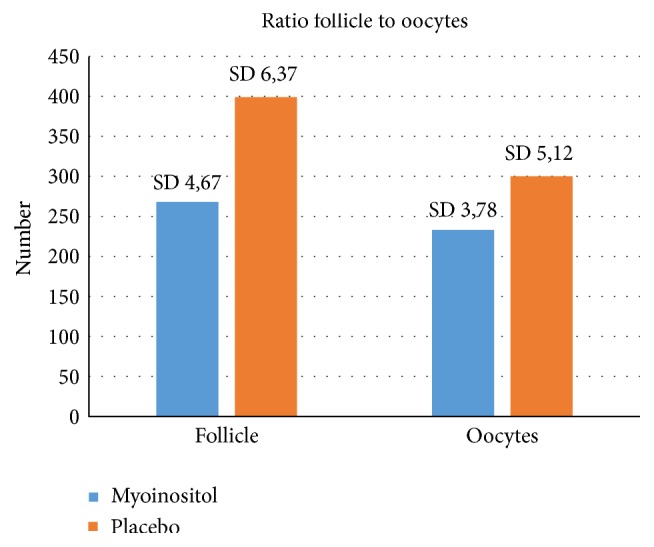
Ratio of follicles and retrieved oocytes. Statistically differences (*p* < 0.05) were observed. The amount of developed follicles was lower in the myoinositol group and in relation to this a higher number of oocytes were retrieved.

Group A (= placebo group) showed a higher amount of retrieved oocytes than group B. Nevertheless the ratio follicle/retrieved oocyte was clearly lower in the myoinositol group (= group B) (*p* < 0.05) (see Figure 1).

**Figure 2:**
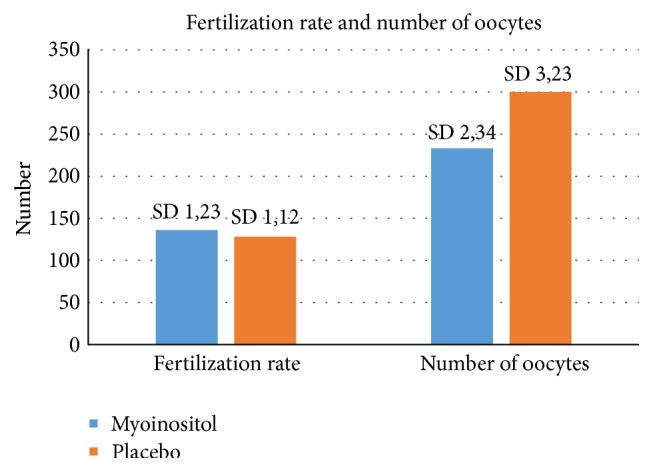
Relation between the myoinositol treatment and the fertilization rate in both groups. Higher rates (*p* < 0.05) were observed in the myoinositol group in comparison to the placebo group.

From the obtained 233 oocytes in the myoinositol group 136 were fertilized whereas only 128 of 300 oocytes were fertilized in the placebo group (*p* < 0.05) (see Figure 2).

**Figure 3:**
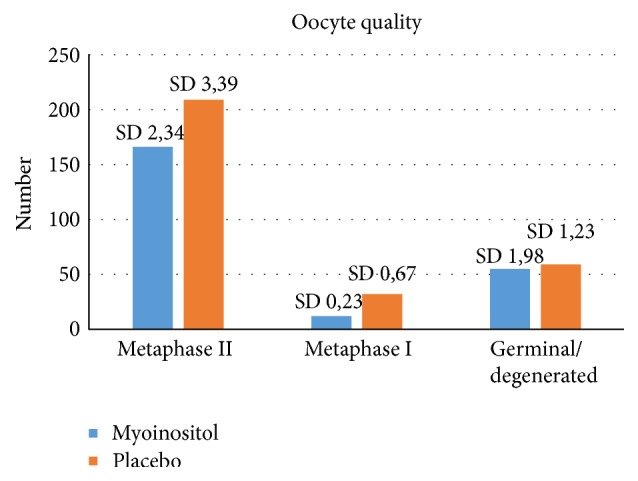
Oocyte quality and treatment with myoinositol or placebo (*p* = 0.06).

In relation to the quality of the oocytes, better data were obtained in the myoinositol group. More metaphase II and I oocytes were retrieved in relation to the total amount of oocytes when compared with the placebo group (*p* > 0.05) (see Figure 3).

**Figure 4:**
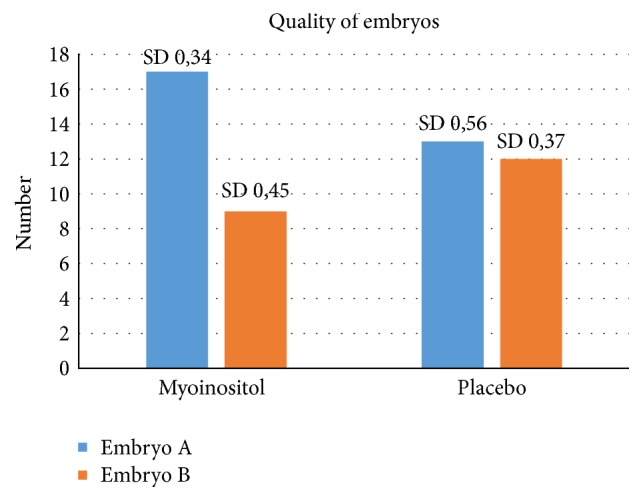
Embryo quality after treatment with myoinositol or placebo. Higher number of embryos with grade I were observed in the myoinositol group (*p* < 0.05).

The last analysis that was performed on the quality of embryos. More embryos of grade I quality were observed in the myoinositol group than in the placebo group (*p* < 0.05) (Figure 4). This difference was, as the analyses done for the fertilization rate and the ratio follicles retrieved oocytes, statistically significant.

The amount of used FSH units was lower in the group of women that received myoinositol. A mean of 1750 units was used (minimum 1350 units and maximum 2250 units whereas the used amount in the placebo group was higher. Mean used units: 1850 with a minimum 1500 and a maximum of 2300 units (*p* > 0.05)). In regard to the amount of stimulation days a significant difference was observed. The myoinositol patients were stimulated with FSH in the mean 9,7 days (±3,3 days) and the placebo group 11,2 days (±1,8 days). This difference was significant (*p* < 0.05).

## 4. Discussion

The Polycystic Ovary Syndrome is one of the most common endocrine disorders affecting women. Insulin resistance and hyperinsulinemia are often found in a high proportion of women with PCOS. A defect in insulin action has been postulated, possibly as a consequence of a deficiency of D-Chiro-Inositol, which is a component of inositol phosphoglycans. Insulin-lowering medications, particularly myoinositol, represent novel therapies for restoring spontaneous ovulation, with a potential positive effect also on human oocyte meiotic maturation. This therapy appears to influence steroidogenesis directly, reducing the androgen production in theca cells. It was shown that inositol administration increases the action of insulin in patients with PCOS, thereby improving ovulatory function and decreasing serum testosterone concentration [12, 23–26].

Studies have shown the positive effects of myoinositol on the outcome of IVF cycles in women with a history of a PCOS. Papaleo et al. [13] could show that in patients with PCOS the treatment with myoinositol and folic acid, but not folic acid alone, reduces germinal vesicles and degenerated oocytes at ovum pick-up without compromising total number of retrieved oocytes. By the same way the amount of stimulation days was, as in our study, shorter in the myoinositol treated women showing a faster response of the ovarian follicles to FSH stimulation due to the myoinositol effects.

On the other side PCOS women have an increased risk of hyper stimulation syndrome [27]. High levels of serum ovarian androgens are associated to a production of elevated serum E2 levels after gonadotropin ovarian stimulation. The study of Papaleo et al. (2009) showed that PCOS patients treated with myoinositol and gonadotropin had a significant reduction in E2 levels after hGC administration [28]. This was related also to the lower number of in vitro fertilization (IVF) cycles cancelled because of high E2 levels (sign of hyper stimulation syndrome).

In addition to this, myoinositol is an important constituent of follicular fluid, playing a key role in both nuclear and cytoplasmic oocyte development. In fact, supplementation with myoinositol in the IVF technique is positively associated to meiotic progression of mouse germinal vesicle oocytes, enhancing intracellular Ca^2+^ oscillation [17]. In human follicular fluids, higher concentrations of MI provide a marker of good-quality oocytes [16, 28, 29].

Beside the increased ratio follicles/retrieved oocytes and the higher fertilization rate in the group of women that used myoinositol, a more important result was the increased number of top-quality (score 1 versus 2) embryos in the myoinositol group. These data suggest that, besides increasing the fertilization rate, myoinositol supplementation has also an effect on the overall quality of the oocyte pool.

Though, additional investigations on larger number of patients are needed to further characterize the impact of myoinositol treatment on oocyte follicular development and oocyte maturation and its implication in stimulation and pregnancy outcomes in IVF procedures.
